# The therapeutic potential of TREM2 in cancer

**DOI:** 10.3389/fonc.2022.984193

**Published:** 2022-09-02

**Authors:** Elysa M. Wolf, Barbara Fingleton, Alyssa H. Hasty

**Affiliations:** ^1^ Department of Molecular Physiology and Biophysics, Vanderbilt University, Nashville, TN, United States; ^2^ Department of Pharmacology, Vanderbilt University, Nashville, TN, United States; ^3^ Veterans Affairs Tennessee Healthcare System, Nashville, TN, United States

**Keywords:** TREM2 (triggering receptor expressed on myeloid cells 2), immunotherapy, tumor associated macrophage (TAM), immunosuppression, tumor infiltrating lymphocyte (TIL)

## Abstract

Cancer continues to be a substantial health concern and a leading cause of death in the United States and around the world. Therefore, it is important to continue to explore the potential of novel therapeutic targets and combinatorial therapies. Triggering receptor expressed on myeloid cells 2 (TREM2) is a transmembrane receptor of the immunoglobulin superfamily that associates with DNAX activation protein (DAP) 12 and DAP10 to propagate signals within the cell. TREM2 has primarily been recognized for its expression on cells in the monocyte-macrophage lineage, with the majority of work focusing on microglial function in Alzheimer’s Disease. However, expansion of TREM2 research into the field of cancer has revealed that epithelial tumor cells as well as intratumoral macrophages and myeloid regulatory cells also express TREM2. In this review, we discuss evidence that TREM2 contributes to tumor suppressing or oncogenic activity when expressed by epithelial tumor cells. In addition, we discuss the immunosuppressive role of TREM2-expressing intratumoral macrophages, and the therapeutic potential of targeting TREM2 in combination with immune checkpoint therapy. Overall, the literature reveals TREM2 could be considered a novel therapeutic target for certain types of cancer.

## Introduction

Triggering receptor expressed on myeloid cells 2 (TREM2) is a transmembrane receptor of the immunoglobulin superfamily that binds an array of ligands including bacteria and polyanionic molecules (1), DNA (2), lipoproteins (3), phospholipids (4) and sulfoglicolipids such as Sulfavant A (5). TREM2 itself does not contain intrinsic signaling capabilities; therefore, it associates with the adaptor proteins DNAX activation protein (DAP) 12 and DAP10; which, upon TREM2-ligand interaction are phosphorylated and propagate signals within the cell (6). TREM2-ligand interaction and subsequent ITAM phosphorylation of DAP12, which is the primary adaptor protein for TREM2, results in activation of Syk, which leads to the phosphorylation of ERK1/2, PLCγ1, and Cbl (7, 8). In contrast, activation of DAP10 results in recruitment of PI3K and activation of Grb2, leading to Akt and ERK signaling respectively (6). While the strength and direction of TREM2 downstream signaling is differentially modulated upon interaction with various ligands, many aspects of TREM2 interaction and the downstream signals propagated remain to be fully understood (7). In addition to signaling through TREM2-ligand interaction and propagation of signals through DAP12 and DAP10, cleavage of TREM2 by a disintegrin and metalloproteinase (ADAM) proteases results in soluble TREM2 (sTREM2), which can act as a signaling molecule. ADAM 10 and 17 cleave human TREM2 at the H157-S158 peptide bond to release the ectodomain of TREM2 (9). Treatment with sTREM2 in *in vitro* studies has resulted in ERK and MAPK14 activation in bone marrow derived macrophages (10) and NF-κB activation in microglia; however, the receptors for sTREM2 remain unidentified (11).

Generally, TREM2 is appreciated for its expression on the surface of cells in the monocyte-macrophage lineage, such as microglia and osteoclasts, with implications for neurodegenerative diseases (12–15) and bone disorders (16, 17). However, more recently, TREM2 has been identified on certain epithelial-derived cancer cells and its expression influences their behavior. This review will focus on the role of TREM2 in cancer, including patient survival data and TREM2 expression in human tumor samples, as well as a discussion of the potentially oncogenic or tumor suppressive roles of TREM2 when expressed by the epithelial tumor cells. In addition, it will provide information on the immunosuppressive environment created by tumor infiltrating immune cells expressing TREM2.

## TREM2 discovery and early implications to human health

TREM2 was first discovered in human monocyte-derived dendritic cells (DCs), where its expression promoted DC survival and upregulation of CCR7, major histocompatibility complex class II, CD86, and CD40 (8). However, TREM2 was first implicated in human health and disease when variants of TREM2 and its adaptor protein DAP12 were identified in families with Nasu-Hakola Disease, which is also known as polycystic lipomembranous osteodysplasia with sclerosing leukoencephalopathy. Patients with Nasu-Hakola Disease are homozygous for loss-of-function mutations in either DAP12 or TREM2, and the disease is characterized by presenile dementia and bone cysts (18–20). With the realization of the importance of TREM2 in neuronal health, investigators have also shown that TREM2 plays a protective function against development of Alzheimer’s Disease. Microglia in the brain express TREM2 and mutations of TREM2 impact its ability to bind ligands, diminish microglial activation, and accelerate progression of Alzheimer’s Disease (21). Up to this point, the majority of work on TREM2 has been conducted in the context of Nasu-Hakola Disease and Alzheimer’s Disease and the role of TREM2 in these settings has recently been reviewed elsewhere (22, 23). However, the importance of TREM2 in cancer has recently come to light, although it is not yet widely studied or understood, hence the focus on cancer for this review.

## TREM2 expression in human tumors and correlations with human survival data

### TREM2 in human tumor samples

Multiple studies have analyzed TREM2 mRNA expression and protein expression in human tumor tissue compared to normal tissue as summarized in **Table 1**. However, the data from different groups are sometimes contradictory even within the same cancers, such as hepatocellular carcinoma (HCC) and gastric cancer. In both of these cases, studies have analyzed TREM2 expression and reported opposite findings despite using similar methods. This highlights the heterogeneity of human tumors as well as the need for further investigation and understanding of the role of TREM2 in cancer.

**Table 1:** TREM2 expression in human tumors.

Cancer type	mRNA expression	Protein expression	Reference
**Hepatocellular Carcinoma**	Decreased (qRT-PCR)	Decreased (WB, IHC)	Tang et al. (24)
	Increased (TCGA)	Increased (IHC)	Esparza-Baquer et al. (25)
	Increased (TCGA)	Increased (IHC)	Cheng et al. (26)
	Increased (scRNAseq)	Increased (IHC)	Zhou et al. (27)
**Gastric cancer**	Increased (qRT-PCR)	Increased (IHC)	Zhang et al. (28)
	not assessed	Decreased (IHC)	Tang et al. (24)
	Increased (TCGA)	NA	Cheng et al. (26)
**Pancreatic cancer**	not assessed	Increased (IHC)	Tang et al. (24)
**Glioma**	Increased (qRT-PCR, TCGA)	Increased (IHC)	Wang et al. (29)
**Glioblastoma multiforme**	Increased (TCGA)	NA	Cheng et al. (26)
**Renal cell carcinoma**	Increased (qRT-PCR)	Increased (WB)	Zhang et al. (30)
**Kidney renal clear cell carcinoma**	Increased (TCGA)	NA	Cheng et al. (26)
**Kidney renal papillary cell carcinoma**	Increased (TCGA)	NA	Cheng et al. (26)
**Kidney chromophobe**	Increased (TCGA)	NA	Cheng et al. (26)
**Colon cancer**	NA	Decreased (IHC)	Kim et al. (31)
	Increased (TCGA)	Increased (IHC)	Cheng et al. (26)
**Head and neck squamous cell carcinoma**	Increased (TCGA)	Increased (IHC)	Cheng et al. (26)
**Uterine corpus endometrial carcinoma**	Increased (TCGA)	NA	Cheng et al. (26)
**Cholangiocarcinoma**	Increased (TCGA)	NA	Cheng et al. (26)
**Prostate adenocarcinoma**	Increased (TCGA)	NA	Cheng et al. (26)
**Bladder urothelial carcinoma**	Increased (TCGA)	NA	Cheng et al. (26)
**Breast cancer**	Increased (TCGA)	Increased (IHC)	Cheng et al. (26)
	Increased (TCGA)	NA	Nalio Ramos et al. (32)
**Cervical squamous cell carcinoma and endocervical adenocarcinoma**	Increased (TCGA)	Increased (IHC)	Cheng et al. (26)
**Thyroid carcinoma**	Increased (TCGA)	NA	Cheng et al. (26)
**Esophageal carcinoma**	Increased (TCGA)	NA	Cheng et al. (26)
**Lung squamous cell carcinoma**	Decreased (TCGA)	Decreased (IHC)	Cheng et al. (26)
**Non-small cell lung cancer**	Decreased (TCGA)	Decreased (IHC)	Cheng et al. (26)
	NA	Increased (FC)	Zhang et al. (33)

qRT-PCR, quantitative real time polymerase chain reaction; TCGA, The Cancer Genome Atlas (transcript level); WB, western blot; IHC, immunohistochemistry; scRNAseq, single cell RNA sequencing; FC, flow cytometry; NA, not assessed.Increased or decreased expression of TREM2 in comparison to normal tissue is indicated for each cancer type at the mRNA and protein expression levels. The method of detection is also noted.

An important first step to understanding the role of TREM2 in cancer was to determine which cell type expresses TREM2 in the tumor microenvironment (TME). A study on TREM2 in HCC observed increased TREM2 expression in HCC samples compared to surrounding normal tissue, and IHC staining revealed the TREM2-expressing cells morphologically resembled macrophages (25). Analysis of single cell sequencing from human HCC livers confirmed this morphological observation as the data demonstrated prominent *TREM2* expression in the macrophages (25). These findings have been corroborated by more recent analysis of single cell RNA sequencing that confirmed specific expression of *TREM2* in macrophages in HCC (27). Similarly, Molgora et al. observed increased TREM2 staining in macrophages, as determined by cell morphology, in 75% of carcinomas from various primary sites compared to normal tissue (34). IHC of primary carcinomas and melanomas demonstrated co-expression of TREM2 with macrophage markers CD163, CD68, MAF-B, CSF1R, and MITF; however, the study did not specify the types of cancer analyzed (34).

Interestingly, analysis of liver, lung, and lymph node metastases originating from ovarian serous and breast carcinoma and colorectal and lung adenocarcinoma by IHC demonstrate specific TREM2^+^ staining within the metastatic nodules and not in the surrounding normal tissue (34). Spatial analysis of TREM2 expression by IHC within tumors shows TREM2^+^ macrophages are primarily localized in the tumor nest in hepatocellular, lung, and pancreatic carcinomas. In other cancers, TREM2^+^ macrophages were found within both the tumor nest and tumor stroma (32). Although these studies indicate TREM2 is expressed on tumor associated macrophages (TAMs), the conclusions of these studies do not exclude that other cell types in the tumor might also express TREM2.

### Human survival data

In a systematic pan-cancer analysis of TREM2 across 33 cancer types, Cheng et al. identified positive and negative associations of TREM2 expression with prognosis in different cancers using data extracted from the TCGA. Kaplan Meier analysis indicated high TREM2 expression was associated with better overall survival in cervical squamous cell carcinoma, endocervical adenocarcinoma, lymphoid neoplasm diffuse large B-cell lymphoma, lung adenocarcinoma, thyroid carcinoma, and skin cutaneous melanoma (26). In contrast, the study found that high TREM2 expression was associated with worse overall survival in lower grade glioma, liver hepatocellular carcinoma, and kidney renal clear cell carcinoma (26). The seeming discrepancy between TREM2 benefitting or worsening patient prognosis in different types of cancer may at first seem puzzling; however, the investigators dove deeper to better understand TREM2 in each cancer type. Gene expression data and protein-level data from IHC demonstrate differences in TREM2 expression between cancer types with the highest expression in glioblastoma multiforme and the lowest in acute myeloid leukemia. IHC implementation to compare TREM2 expression between normal and tumor tissue showed increased TREM2 expression in the tumor tissue compared to normal tissue in many cancers. However, in other cancers, such as lung squamous cell carcinoma, TREM2 staining was moderate in the normal tissue and weak in the tumor tissue. These deviations in expression of TREM2 indicate that TREM2 may serve distinct roles and may exhibit differing levels of influence in distinct types of cancer. By probing associations between TREM2 and tumor mutation burden, immune scores, tumor stage, DNA methylation, and infiltration of immune cells the investigators highlight the heterogeneity among tumor types. TREM2 may interact differently with each of these factors, thus summing to differing prognoses in patients.

While this pan-cancer analysis is a great resource, there are also previous smaller studies that individually confirm or contradict the findings from the pan-cancer analysis. In agreement with this study, Wang et al. previously demonstrated an association between high TREM2 and worse overall survival in glioma (29). This same association has also been shown in gastric cancer (28), colorectal cancer (CRC) (34), triple negative breast cancer (34) and luminal breast cancer (32), suggesting TREM2 contributes to oncogenic activity in these cancer types. However, in disagreement with the systematic pan-cancer analysis, a previously published study by Tang et al. demonstrated increased TREM2 expression correlates with better overall survival in HCC (24), indicating TREM2 may contribute to tumor suppressing activity in HCC. The discrepancy between the two studies may be explained by use of data from two distinct cohorts of patients. The pan-cancer analysis utilized the TCGA while Tang et al. evaluated a cohort of 250 patients with HCC whose surgically resected samples and survival data were obtained and analyzed by the investigators. Furthermore, Tang et al. collected primary tumor, matched non-tumor liver tissue and venous metastasis from the subjects, which indicates all tumor specimens had metastasized, thus skewing the data set. Another more recent study demonstrated high levels of TREM2^+^ TAMs predicted worse overall survival in both lung adenocarcinoma and lung squamous cell carcinoma, which is also in disagreement with the pan-cancer analysis. This may be accounted for by the specific attention of some groups to macrophage expression of TREM2 rather than overall expression of TREM2.

As discussed in the sections below, the *in vitro* and *in vivo* data from mouse studies do not always align with the human survival data. In the data covered in this review, this is the case specifically in the models of CRC (31), which may be due to the inability of the mouse model to fully recapitulate the human disease. However, this discrepancy could also be related to the method of *TREM2* expression analysis in these Kaplan Meier plots. The data used to generate these plots originate from bulk RNA sequencing. Tumors are comprised of a heterogeneous milieu of cells and *TREM2* can be expressed by the cancer cells or by other immune cell populations such as TAMs. Therefore, the Kaplan Meier plots may be useful as a starting point to evaluate overarching trends, but not as useful for the purpose of delineating the specific role of *TREM2* within the epithelial tumor cells versus the immune cells in the TME. As we learn more about the roles of TREM2 expressed by differing cell types, the utilization of scRNAseq may be critical to advancing our knowledge and understanding of TREM2 in cancer.

## TREM2 in cancer progression

Due to the heterogeneous cellular composition of tumors, the contribution of different proteins on the various cell types complicates therapeutic strategies. When considering TREM2 as a potential therapeutic target, it is critical to understand its functions and properties in different contexts and to consider the cell-type expressing TREM2. The emerging body of literature on the subject of TREM2 expression by the epithelial tumor cells is seemingly contradictory, with some studies suggesting TREM2 contributes to tumor suppressive activity and other studies suggesting it supports oncogenic activity. However, the literature consistently reports that TREM2 expression by immune cells creates an immunosuppressive environment that allows the cancer cells to thrive. In subsequent sections, we summarize what is known about the immunosuppressive and oncogenic roles of TREM2 and suggest areas for future investigation.

## TREM2 contributes to either tumor suppressing or oncogenic activity in different types of cancer

### The case for TREM2 contributing to oncogenic activity

Data on TREM2 expression in epithelial tumor cells is still limited, but two studies, one in renal cell carcinoma (RCC) and one in glioma have been conducted that point to an oncogenic role of TREM2. The RCC study utilized the ACHN and Caki-2 RCC cell lines (30) and the glioma study used the U87 and U373 glioma cell lines (29). In both studies, silencing of *TREM2* resulted in decreased cell proliferation and increased apoptosis *in vitro* and decreased tumor volume *in vivo* with subcutaneous cell injection models (29, 30). Additionally, in the RCC model, silencing of *TREM2 in vitro* resulted in a decrease of *Bcl2*, a regulator of apoptosis, an increase in the apoptosis genes *Bax* and *Casp3*, and a decrease in the proliferation marker PCNA, measured at both the gene and protein expression levels (30). In the glioma study, silencing of *TREM2* led to a decrease in cell adhesion as well as decreases in the migratory and invasive capacities of both cell lines (29). This work suggests epithelial TREM2 contributes to oncogenic activity in the context of RCC and glioma.

### The case for TREM2 contributing to tumor suppressing activity

*In vitro* and *in vivo* studies suggest that TREM2 may contribute to tumor suppressing activity in CRC and HCC. Using HT29 CRC cells, Kim and colleagues demonstrated that antibody-mediated *TREM2* neutralization resulted in increased cell proliferation, induction of the S phase of the cell cycle, increased cell migration, and increased invasive capacity. Conversely, *TREM2*-overexpressing MC38 CRC cells resulted in decreased tumor volume following subcutaneous injection in mice (31). Together these data suggest that epithelial TREM2 may support tumor suppressing activity in CRC. Similarly, in a mouse model of HCC, knockdown of *TREM2* resulted in increased cell viability, increased migratory and invasive capacities, as well as decreased epithelial markers with an increase in mesenchymal markers (24). Subcutaneous injection of cells with *TREM2* knockdown resulted in increased tumor volume suggesting TREM2 may also contribute to tumor suppressing activity in HCC. To further support this conclusion, the same study found that overexpression of TREM2 resulted in opposing results from the knockdown conditions (24). Another study of HCC utilized carcinogen-induced models of HCC in TREM2 deficient mice to probe the function of TREM2 in HCC. The investigators found that mice globally-deficient for TREM2, thus not expressing TREM2 on the epithelial tumor cells or any other cell type within the TME, developed an increased number of tumors of all sizes following carcinogen (DEN: diethylnitrosamine) administration and also developed an increased number of as well as larger tumors in fibrosis associated HCC models (25). While these data overall support TREM2 contributing to tumor suppressing activity in HCC and CRC, mechanistic conclusions are clouded by the global deletion of TREM2. All studies are summarized in **Table 2**.

**Table 2:** Summary of mouse studies with supporting evidence for TREM2 contributing to tumor suppressing or oncogenic activity in different types of cancer.

	Cancer type	Cancer model	Phenotype	Reference
Tumor Suppressive Activity	HCC	s.c. TREM2 KD	Increase tumor size	Tang et al. (24)
		s.c. TREM2 OE	Decrease tumor size	
		i.v. (tail vein) TREM2 OE	Suppress lung metastasis	
	HCC	DEN-induced carcinogenesis, *Trem2* ^-/-^ mice	Increase tumor number	Esparza-Baquer et al. (25)
		DEN/CCl_4_-induced carcinogenesis, *Trem2* ^-/-^ mice	Increase tumor number	
		TAA-Induced carcinogenesis, *Trem2* ^-/-^ mice	Increase tumor volume	
	CRC	s.c. TREM2 OE	Decrease tumor volume	Kim et al. (31)
Oncogenic activity	Glioma	s.c. TREM2 KD	Decrease tumor volume	Wang et al. (29)
	RCC	s.c. TREM2 KD	Decrease tumor volume	Zhang et al. (30)

HCC, hepatocellular carcinoma; CRC, colorectal cancer; RCC, renal cell carcinoma; s.c., subcutaneous injection of cancer cells; i.v., intravenous; KD, knockdown; OE, overexpression; DEN, diethylnitrosamine; CCl_4_, carbon tetrachloride; TAA, thioacetamide.

## Cancer-associated fibroblast TREM2 expression may modulate paracrine signaling and tumorigenicity

Perugorria et al. previously showed that TREM2 can be expressed in activated hepatic stellate cells (HSCs) in the context of liver injury, which modulates toll like receptor-mediated inflammation (35). Therefore, following the observation that TREM2 deficient mice developed an increased number of tumors in carcinogen-induced models of HCC, the investigators interrogated how TREM2 expressed by HSCs would impact tumorigenicity. The researchers evaluated tumorigenicity utilizing a hanging droplet liver cancer spheroid growth assay. Consistent with findings from the *TREM2* deficient carcinogen-induced models of HCC, conditioned media from *TREM2*-overexpressing HSCs suppressed spheroid growth (25). Further analysis revealed that *TREM2* overexpression in HSCs attenuated expression of multiple canonical Wnt ligands, which may have contributed to spheroid growth suppression. Although these data are limited to HCC, they open up the prospect that activated cancer-associated fibroblasts in other cancer types may express TREM2, and in doing so may modulate paracrine signaling to the surrounding cells. However, it is of note that these studies were only conducted *in vitro* and thus, future studies should include further analysis of cancer associated fibroblasts in both HCC and other types of cancer both *in vitro* and *in vivo*.

## TREM2 expression and functions within the immune cell populations of the TME

### Expression of TREM2 by myeloid cells in the TME creates an immunosuppressive environment

CD8^+^ cytotoxic T lymphocytes are key immune cells for controlling tumor growth by killing cancer cells that express major histocompatibility complex class I molecules. However, immunosuppressive crosstalk between cancer cells and other cell types within the TME, such as cancer-associated fibroblasts, regulatory T cells, and M2-polarized macrophages, can suppress the effector functions of CD8^+^ T cells (36). Given that analysis of human tumor samples from various primary carcinomas including those of skin, liver, lung, breast, bladder, colon, stomach, pancreas, and kidney contain TREM2^+^ macrophages in 75% of samples (34), there is reason to consider that TREM2 expression contributes to the immunosuppressive phenotype. In settings of infection, TREM2 enhances phagocytosis and reduces pro-inflammatory cytokine secretion by macrophages, thus serving an immunoregulatory role (7). Current knowledge suggests that TREM2 expression on cells of the monocyte-macrophage lineage may also serve an immunoregulatory role in cancer, creating an immunosuppressive environment. Recent work by Drake and colleagues uncovered a tumor-specific C1Q^+^TREM2^+^APOE^+^ macrophage population in clear cell renal carcinoma associated with post-surgical disease recurrence for patients (37). This suggests that TREM2 expression in tumor-specific macrophages is associated with a pro-tumorigenic environment.

TREM2 has primarily been shown to be expressed on the surface of cells in the monocyte-macrophage lineage, including microglia (38), osteoclasts (39), and other macrophages such Kupffer cells (40) and lipid associated macrophages (LAMs) in adipose tissue (41). Studies have used scRNAseq to understand how *TREM2* deficiency impacts the myeloid compartment in the TME as well as tumor progression. One study identified two populations of tumor-infiltrating myeloid suppressive cells that express TREM2 in a subcutaneous MCA-205 fibrosarcoma model: a TAM population and a myeloid regulatory cell population (42). A second study employed a subcutaneous MCA/1956 model in *Trem2* ^+/+^ and *Trem2* ^-/-^ mice to understand how TREM2 deficiency impacts the myeloid compartment. The initial analysis of all CD45^+^ cells demonstrated that *Trem2* was expressed on all macrophage clusters albeit at varying levels, but no TREM2 was detected in DCs or lymphoid cells. Further re-clustering of macrophages identified specific macrophage clusters with high expression of *Trem2* in the *Trem2* ^+/+^ mice. The presence of these specific macrophage clusters was significantly diminished in the *Trem2* ^-/-^ mice suggesting that TREM2 may be responsible for sustaining these populations of macrophages (34). This demonstrates that TREM2 deficiency impacts the restructuring of the myeloid cell compartment within a tumor.

Following these observations, tumor growth was evaluated in *Trem2* ^-/-^ and *Trem2* ^+/+^ mice utilizing a subcutaneous injection model with either MCA-205 (42) or MCA/1956 (34) sarcoma cell lines. Tumor growth attenuation was observed in the *Trem2* ^-/-^ mice compared to the *Trem2* ^+/+^ mice with both cell lines (34, 42). Likewise, Molgora et al. observed tumor growth attenuation in a MC38 CRC subcutaneous model and an orthotopic mammary model in *Trem2* ^-/-^ mice (34). The TREM2 expression profile on the cancer cells injected in these studies was not reported. Both studies attribute the reduction in tumor growth to the lack of TREM2 expression on the immune cells and thus a reduced ability of the immune cells to create an immunosuppressive environment. It is important to note, given the differential effects by tumor type above, that both of these papers utilized sarcoma models in their scRNAseq studies, which are mesenchymal rather than epithelial derived tumors. scRNAseq was not performed for the MC38 CRC subcutaneous model or the orthotopic mammary model.

More recent studies utilizing scRNAseq have begun to elucidate and characterize TREM2-expressing macrophages in epithelial tumors. TREM2^+^ macrophages identified in the lungs of a mouse mammary tumor model demonstrated a gene expression profile akin to LAMs with positive enrichment for pathways associated with cholesterol and lipid metabolism (43). Interestingly, these LAMs are increased in the lungs of mammary tumor-bearing mice compared to non-tumor bearing mice and are enriched for protumorigenic pathways related to negative regulation of T-cell responses, epithelial-mesenchymal transition, and endothelial cell proliferation (43). An increased presence of these LAMs in the lungs at a premetastatic time point suggests an immunosuppressive preparation of the metastatic niche. In complement to this study, a TREM2-expressing macrophage subpopulation in HCC patient tissues were reported to resemble hepatic LAMs with upregulation of immunosuppressive pathways such as Treg recruitment and angiogenesis stimulation (27). TREM2^+^ TAMs in non-small cell lung cancer (NSCLC) patient tissues were also enriched for fatty acid metabolism and protumorigenic pathways (33).

While there is much to learn about the role of TREM2 in cancer, some key studies indicate that TREM2 is involved in suppressing the function of CD8^+^ T cells as well as inhibiting their proliferation, which would argue for myeloid-targeted inhibition of TREM2 at least in some cancers.

### Impairment of CD8^+^ T cells by TREM2^+^ myeloid cells and recruitment of T regulatory cells may provide a mechanistic link for immunosuppression

Although T cells don’t express TREM2, it is possible they can be impacted by TREM2 expression on other cell types within the TME. Subcutaneous MCA/1956 tumors in mice deficient for TREM2 displayed an increase in CD8^+^ T cells as a percent of all tumor infiltrating T cells compared to wild-type mice. These CD8^+^ T cells were deemed activated based on PD-1 expression and tumor growth was restrained in the TREM2 deficient mice. Administration of an anti-CD8 monoclonal antibody in both *Trem2* ^+/+^ and *Trem2* ^-/-^ mice accelerated tumor growth compared to the controls (34). In another study utilizing subcutaneous injection of MCA-205 fibrosarcoma cells in *Trem2* ^-/-^ mice, the investigators not only observed a reduction in tumor growth, but also an expansion of the natural killer and cytotoxic T cell population accompanied by a decrease in dysfunctional CD8^+^ T cells (42). Therefore, these data suggest that tumor growth attenuation in the TREM2 deficient conditions is mechanistically linked to the activation of CD8^+^ T cells. Additionally, the data suggest that the presence of TREM2^+^ cells in the tumor stroma contributes to the suppression of these cytotoxic T lymphocytes and their ability to control tumor growth.

In addition to the impaired effector functions of cytotoxic T lymphocytes, the proliferation of these cells may be diminished by the expression of TREM2 on cells in the TME. Bone marrow derived DCs were induced to express TREM2 by culturing with conditioned media from 3LL lung cancer cells. Yao et al. found that T cells co-cultured with *Trem2* ^+^ DCs exhibited lower levels of proliferation compared to T cells co-cultured with *Trem2* deficient DCs or a combination of TREM2^+^ DCs with anti-TREM2 mAb (44). In another recent study, N9 macrophages either positive or deficient for TREM2 were co-cultured with CD8^+^ T cells. The results demonstrate that co-culture of the CD8^+^ T cells with *Trem2* ^+/+^ N9 cells suppresses T cell proliferation in a manner at least comparable to treatment with transforming growth factor β (42).

These observations have also been corroborated in human tumor tissue. Assessment of fresh NSCLC patient samples by flow cytometry revealed that tumors with high TREM2^+^ TAM infiltration exhibited a decrease in CD8^+^ T cells expressing CD107a, perforin 1, and tumor necrosis factor-α suggesting a decrease in effector function (33). This is corroborated in *ex vivo* studies where co-culture of CD8^+^ T cells with TREM2^+^ TAMs also resulted in decreased T cell proliferation and reduction of CD107a, perforin 1, and tumor necrosis factor-α production (33).

In addition to reduction of CD8^+^ T cell proliferation and effector function, two independent studies in NSCLC and HCC suggest immunosuppressive TREM2^+^ TAMs are also involved in recruiting T regulatory cells (Tregs). Implementation of CellPhoneDB, which infers cell-cell communication networks from scRNAseq data, to HCC patient samples revealed interaction of TREM2^+^ LAM-like cells with Tregs *via* the CCL20/CXCL9/CXCL10/CXCL12-CXCR3 axis, suggesting recruitment of Tregs by the TREM2^+^ LAM-like cells by means of migration-related chemokines (27). Assessment of cell-cell interactions in NSCLC patient sample scRNAseq data identified an interaction between IL1β and IL1R from TREM2^+^ TAMs and FOXP3^+^ Tregs, respectively (33). Further, NSCLC samples with high TREM2^+^ TAMs exhibited an increase in transforming growth factor-β-expressing FOXP3^+^ Tregs by flow cytometry (33), and TREM2^+^CD163^+^ macrophages were found to colocalize with FOXP3^+^ Tregs within HCC tumors (27). These data suggest decreased effector functions of CD8^+^ T cells may be due in part to the infiltration and function of Tregs. The interplay between stromal cells in the TME is complex, and while it is unlikely that TREM2 impacts the tumor *via* a single mechanism, these data indicate that key mechanisms are through suppression of cytotoxic T lymphocytes and recruitment of regulatory T cells.

### TREM2 expression may confer resistance to immune checkpoint therapy

As noted, TREM2 expression by myeloid cells can impact T cell activation and proliferation; thus, it is not surprising that TREM2 may be able to serve as a biomarker for tumor burden and high TREM2 expression may confer resistance to immune checkpoint therapy (ICT). Yao et al. found an increase of TREM2 positive monocytes in the peripheral blood of lung cancer patients and in both the peripheral blood and lungs of tumor-bearing mice (44). Furthermore, TREM2 expression on macrophages in lung cancer patient samples increased with both pathological staging of disease as well as degree of lymph node metastasis (44). Lung cancer patients that responded to chemotherapy with a reduction of tumor burden displayed a decrease in TREM2 positive monocytes in the peripheral blood. Additionally, lung cancer patients that underwent surgical tumor resection, and thus had a reduced tumor burden, also displayed a decrease in TREM2 positive monocytes in the peripheral blood (44).

NSCLC patients with high TREM2^+^ TAM infiltration had a lower objective response rate to ICT compared to patients with low numbers of TREM2^+^ TAMs and were more likely to experience tumor progression following PD-1 blockade (33). Analysis of scRNAseq data from melanoma patients divided into subgroups of responders and non-responders to ICT revealed a significant enrichment of macrophages with high expression of TREM2 in non-responders, implying that macrophage cell populations with high expression of TREM2 may precipitate ICT resistance (45). Overall, these studies demonstrate an upregulation of TREM2 on TAMs with increasing disease severity and in patients non-responsive to ICT.

### TREM2 modulation enhances anti-PD-1 therapy

Molgora et al. probed whether neutralization of TREM2 in combination with ICT improves tumor response to treatment (34). The investigators first established that treatment with αPD-1 in TREM2 deficient mice led to further tumor control and regression in sarcoma and CRC models. Furthermore, subsequent treatment of wild-type mice with combined αTREM2 and αPD-1 mAb led to complete reduction of tumor burden in all mice tested. This demonstrates that deficiency of TREM2 or treatment with anti-TREM2 mAb augments the efficacy of αPD-1 ICT.

Currently, a Phase I clinical trial (ClinicalTrials.gov identifier: NCT04691375) for a TREM2 mAb administered either as a single agent or in combination with pembrolizumab (αPD-1) is underway. The subjects in this clinical trial have locally advanced and/or metastatic solid tumors that are refractory or relapsed to standard of care treatment (46). This specific TREM2 mAb, PY314, is a depleting antibody designed to deplete tumor associated macrophages expressing TREM2. This study was initiated in October of 2020 and the estimated completion date is October 2023 (46)

## Conclusion and future directions

**Figure 1:**
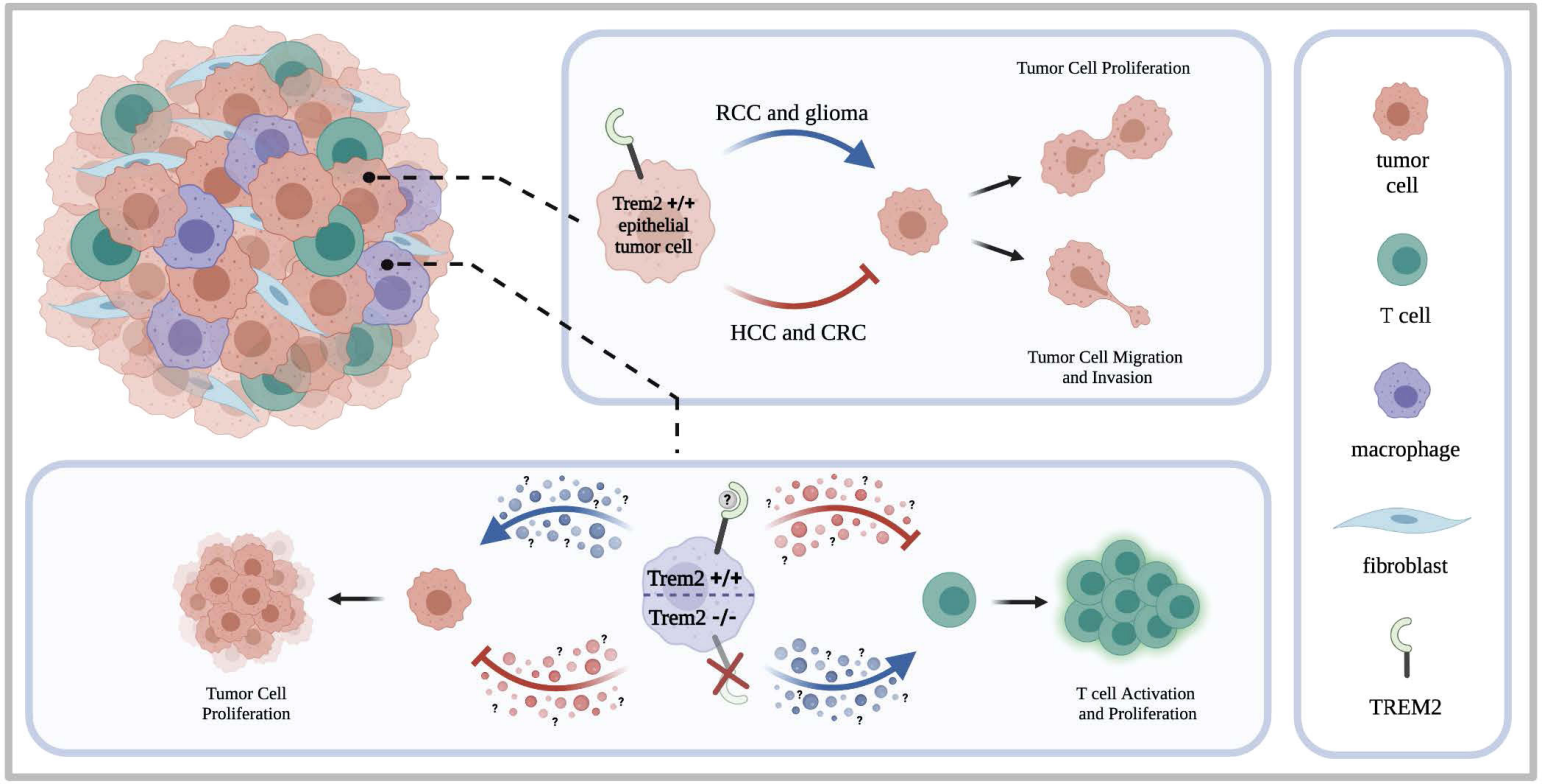
TREM2 in heterogeneous tumors. Tumors are composed of a heterogeneous makeup of cells including the tumor cells, tumor associated macrophages, T cells, and tumor associated fibro blasts. Studies have shown that TREM2 can be expressed by both epithelial tumor cells and infiltrating immune cells such as macrophages. When expressed by the epithelial tumor cells, studies indicate that TREM2 supports oncogenic activity in renal cell carcinoma and glioma but contributes to tumor suppressing activity in renal cell carcinoma. Thus, contributing to oncogenic or tumor suppressing activity, TREM2 can enhance or inhibit tumor cell proliferation and tumor cell migration and invasion. When expressed by infiltrating immune cells such as macrophages, the literature indicates that TREM2 expressing immune cells contribute to creating an immunosuppressive environment by inhibiting T cell activation and proliferation. Consequently, tumor cell proliferation is then enhanced. When TREM2 is knocked out on the tumor infiltrating immune cells, increased T cell activation and proliferation is observed accompanied by a decrease in tumor growth.

When considering novel therapeutic targets, it is important to consider how different cell types may respond to therapy and thus impact the patient’s overall response and outcome. As covered in this review and summarized in **Figure 1**, TREM2 is expressed by multiple cell types within the TME. TREM2 may have tumor cell intrinsic functions in addition to its role in stromal cells and fibroblasts that could be tumor suppressive or oncogenic depending on the type of cancer. Therefore, moving forward it is imperative that we better understand the mechanisms by which TREM2 contributes to tumor suppressive or oncogenic activity in the cancer types discussed in this review. Within this review, discrepancies are unsurprisingly found between *in vitro* studies, mouse models, and human data. As future studies are conducted, use of *in vitro* methods and cell lines should be accompanied with complementary *in vivo* and human data to ensure the rigor of the data. Additionally, we need to understand the tumor cell intrinsic role of TREM2 in more types of solid cancers such as breast cancer. This understanding is key since anti-TREM2 mAb treatment is already undergoing clinical testing for a variety of solid tumor types. Overall, the data indicate that high expression of TREM2 on cells of the monocyte-macrophage lineage creates an immunosuppressive environment in which T cells are less activated and their proliferation is suppressed. Therefore, this pro-tumoral role of TREM2 is the current prevailing opinion in the literature. Thus, inhibition or blockade of TREM2 may be an effective therapeutic strategy. However, depending on the role in tumor cells, blockade of TREM2 may still be clinically unfavorable. With these varying results, it is of the utmost importance to continue to uncover the role of TREM2 in cancer.

## Author contributions

EW wrote the first draft of the manuscript. BF and AH edited the manuscript. All authors approve of the final submitted version.

## Funding

EW was supported by a training grant from the National Institute of Allergy and Infectious Diseases (T32AI38932). AH is supported by a Career Scientist Award from the Veterans Affairs (IK6 BX005649).

## Conflict of interest

The authors declare that the research was conducted in the absence of any commercial or financial relationships that could be construed as a potential conflict of interest.

## Publisher’s note

All claims expressed in this article are solely those of the authors and do not necessarily represent those of their affiliated organizations, or those of the publisher, the editors and the reviewers. Any product that may be evaluated in this article, or claim that may be made by its manufacturer, is not guaranteed or endorsed by the publisher.
